# Hypoxia-induced preadipocyte factor 1 expression in human lung fibroblasts through ERK/PEA3/c-Jun pathway

**DOI:** 10.1186/s10020-021-00336-w

**Published:** 2021-07-06

**Authors:** Wun-Hao Cheng, Chia-Ling Chen, Jing-Yun Chen, Chien-Huang Lin, Bing-Chang Chen

**Affiliations:** 1 Gradual Institute of Medical Sciences, College of Medicine, Taipei Medical University, 250 Wu-Hsing Street, Taipei, 11031 Taiwan; 2 Division of Pulmonary Medicine, Department of Internal Medicine, School of Respiratory Therapy, Wan Fang Hospital, Taipei Medical University, 250 Wu-Hsing Street, Taipei, 11031 Taiwan; 3 Division of Thoracic Medicine, Department of Internal Medicine, School of Medicine, College of Medicine, Taipei Medical University, Taipei, Taiwan; 4 School of Respiratory Therapy, College of Medicine, Taipei Medical University, Taipei, Taiwan

**Keywords:** Hypoxia, Pref-1, ERK, PEA3, AP-1, Human lung fibroblasts

## Abstract

**Background:**

Several studies have reported that hypoxia plays a pathological role in severe asthma and tissue fibrosis. Our previous study showed that hypoxia induces A disintegrin and metalloproteinase 17 (ADAM17) expression in human lung fibroblasts. Moreover, preadipocyte factor 1 (Pref-1) is cleaved by ADAM17, which participates in adipocyte differentiation. Furthermore, Pref­1 overexpression is involved in tissue fibrosis including liver and heart. Extracellular signal-regulated kinase (ERK) could active downstram gene expression through polyoma enhancer activator 3 (PEA3) phosphorylation. Studies have demonstrated that PEA3 and activator protein 1 (AP-1) play crucial roles in lung fibrosis, and the Pref-1 promoter region contains PEA3 and AP-1 binding sites as predicted. However, the roles of ERK, PEA3, and AP-1 in hypoxia-stimulated Pref-1 expression in human lung fibroblasts remain unknown.

**Methods:**

The protein expression in ovalbumin (OVA)-induced asthmatic mice was performed by immunohistochemistry and immunofluorescence. The protein expression or the mRNA level in human lung fibroblasts (WI-38) was detected by western blot or quantitative PCR. Small interfering (si) RNA was used to knockdown gene expression. The collaboration with PEA3 and c-Jun were determined by coimmunoprecipitation. Translocation of PEA3 from the cytosol to the nucleus was observed by immunocytochemistry. The binding ability of PEA3 and AP-1 to Pref-1 promoter was assessed by chromatin immunoprecipitation.

**Results:**

Pref-1 and hypoxia-inducible factor 1α (HIF-1α) were expressed in the lung sections of OVA-treated mice. Colocalization of PEA3 and Fibronectin was detected in lung sections from OVA-treated mice. Futhermore, Hypoxia induced Pref­1 protein upregulation and mRNA expression in human lung fibroblasts (WI­38 cells). In 60 confluent WI-38 cells, hypoxia up-regulated HIF-1α and Pref-1 protein expression. Moreover, PEA3 small interfering (si) RNA decreased the expression of hypoxia-induced Pref­1 in WI­38 cells. Hypoxia induced PEA3 phosphorylation, translocation of PEA3 from the cytosol to the nucleus, PEA3 recruitment and AP-1 binding to the Pref­1 promoter region, and PEA3-luciferase activity. Additionally, hypoxia induced c-Jun-PEA3 complex formation. U0126 (an ERK inhibitor), curcumin (an AP­1 inhibitor) or c-Jun siRNA downregulated hypoxia-induced Pref-1 expression.

**Conclusions:**

These results implied that ERK, PEA3, and AP­1 participate in hypoxia­induced Pref­1 expression in human lung fibroblasts.

## Introduction

Asthma is an allergic airway disease characterized by airway inflammation, epithelial apoptosis, and airway remodeling (Ahmad 2012; Hough 2020). A study showed that approximately 3.7% of patients with asthma developed severe asthma (Hekking 2015). Severe asthma is a difficult-to-control airway disease; it requires inhalation of high doses of corticosteroids to relieve symptoms (Thomson 2014). Airway fibrosis occurs when fibroblasts differentiate into α-smooth muscle actin myofibroblasts with massive deposition of extracellular matrix, including fibronectin and collagen I (Bergeron et al. 2010; Hoshino et al. 1998; Brewster 1990). Numerous studies have shown that extracellular matrix deposition and fibrosis are correlated with asthma severity (Bergeron et al. 2010; Chetta 1997; Little 2002).

Hypoxia contributes to the fibrosis of several organs (i.e., the kidney, liver, and lung) (Tanaka 2016; Rosmorduc and Housset 2010; Chai 2018; Senavirathna 2018). Hypoxia also exacerbates airway remodeling (Kang 2020; Braun 2018; Guo et al. 2015). Furthermore, hypoxia could damage airway epithelium and promote lung inflammation through hypoxia-inducible factors (HIFs) (Ahmad 2012; Page et al. 2021). HIF-α is a key transcript factor produced in response to hypoxia in cancer, fibrosis, and respiratory inflammation disease (Page et al. 2021; Talks 2000; Bahrami et al. 2018). In contrast, airway inflammation and fibroblast proliferation could cause airway remodeling, leading to tissue hypoxia (Ahmad 2012; Polosukhin 2007; Kostakou, 2019). Thus, hypoxia plays a major role in airway fibrosis.

Preadipocyte factor 1 (Pref-1), a transmembrane protein, is processed to the soluble protein by A disintegrin and metalloproteinase 17 (ADAM17) (Wang et al. 2006). Pref-1 was originally found to be responsible for adipogenesis inhibition through Rac1-ERK pathway in preadipocytes (Wang et al. 2010). Moreover, soluble Pref-1 is involved in many organ fibrosis or cell differentiation (i.e., osteogenesis, heart, and liver fibrosis) (Wang and Sul 2009; Zhu 2012; Tschope and Diez 2019). hypoxia-induced soluble Pref-1 could facilitate tumorigenicity and clonogenicity of neuroblastoma cells (Kim et al. 2009a). However, the role of Pref-1 in human lung fibrosis remains unclear.

Polyoma enhancer activator 3 (PEA3) is one of the erythroblast transformation specific (ETs) domain transcription factor superfamily. PEA3 could activate gene expressions through the ETs domain to recognize GGAA/T core motif in the promoter (Kandemir et al. 2020). Previous studies showed that PEA3 played a crucial role in cancer metastasis and fibrotic gene expression (Kandemir et al. 2020; Warburton et al. 2013). The extracellular signal-regulated kinase (ERK)-induced PEA3 phosphorylation mediate gene expression in collaboration with other transcription factors (O'Hagan et al. 1996; Ratovitski 2010; Iguchi 2000). According to the National Center for Biotechnology Information (NCBI) database, the PEA3 binding sequence existed in the Pref-1 promoter. However, the role of PEA3 in hypoxia-induced Pref-1 needs clarification.

The activator protein 1 (AP-1) transcript factor is a heterodimer composed of c-Jun and c-Fos. AP-1 could regulate gene expressions in cell growth, cell differentiation, and apoptosis (You et al. 2010). Furthermore, AP-1 increased profibrotic protein expressions in lung fibrosis (Weng 2014). Hypoxia could induce diabetic retinopathy through the activation of AP-1 (You et al. 2010). In hepatocellular carcinoma cells, PEA3 interacted with AP-1 to regulate *IL-8*/*CXCL8* gene expression (Iguchi 2000). However, hypoxia-induced Pref-1 expression through the ERK/PEA3/AP-1 pathway remains unclear. We aimed to investigate the mechanism of hypoxia-induced Pref-1 expression in human lung fibroblasts.

## Materials and methods

### Materials

Antibodies specific for Pref-1, HIF-1α, and phospho-serine, secondary antibodies against IP detection reagent, Alexa Fluor-488 and Alexa Fluor-555 were purchased from Abcam (Cambridge, MA, USA). An antibody specific for c-Jun Ser63 was purchased from Cell Signaling Technology (Danvers, MA, USA). Antibodies specific for PEA3, c-Jun, horseradish peroxidase (HRP)-linked antibodies, including anti-goat immunoglobulin G (IgG), anti-rabbit IgG, and anti-mouse IgG antibodies, were obtained from Santa Cruz Biotechnology (Dallas, CA, USA). A luciferase assay kit was purchased from Promega (Madison, WI, USA). The human PEA3 luciferase reporter was a obtained from Peter Hollenhorst (Watertown, MA, USA). Furthermore, α-tubulin antibody, fetal bovine serum, control small interfering RNA (siRNA) (scrambled), c-Jun siRNA and PEA3 siRNA were purchased from Sigma-Aldrich (St. Louis, MO, USA). Lipofectamine 3000 reagent, minimum essential medium (MEM), penicillin, and streptomycin were acquired from Invitrogen Life Technologies (Carlsbad, CA, USA). A Novolink Max Polymer Detection System was purchased from Leica (Wetzlar, Germany).

### Cell culture

Human lung fibroblast (WI-38) cells, purchased from American Type Culture Collection (Manassas, VA, USA), were grown in MEM supplemented with 10% fetal calf serum, penicillin G (100 U/mL), streptomycin (100 µg/mL), and MEM nonessential amino acids. WI-38 cells were maintained in a humidified 37 °C incubator with 5% CO_2_. After reaching 60% or 90% confluence, cells were seeded onto 12-well plates for the transfection and luciferase reporter assay, onto 6-cm dishes for Western blot analysis, and onto 10-cm dishes for the immunoprecipitation assay.

### Animals

Female C57BL/6 mice aged 5–6 weeks were obtained from BioLASCO (Taipei, Taiwan). All animal protocols were approved by the Animal Ethics Committee of Taipei Medical University (approval no. LAC-2016-0361 and LAC-2019-0042).

### Ovalbumin-induced animal model of airway fibrosis

For sensitization, on days 1, 7, and 14, C57BL/6 mice were intraperitoneally injected with 200 μL of 50 μg ovalbumin (OVA) emulsified in 2 mg of aluminum hydroxide. From day 21, 8- to 10-week-old C57BL/6 mice were challenged with aerosolized 5% OVA in phosphate-buffered saline (PBS) or PBS alone for 9 weeks. The frequency of the OVA challenge was twice weekly. After the final OVA aerosol challenge, C57BL/6 mice were sacrificed, and the lung tissue of mice was analyzed with further experiments.

### Hypoxia exposure

WI-38 cells were treated with a 1% O_2_ at 37 °C chamber and flushed with a gas mixture of 5% CO_2_ and 95% N_2_ using a ProOx model 110 oxygen regulator purchased from BioSpherix (New York, NY, USA).

### Western blot analysis

Protein extraction and Western blot analysis were performed as previously described (Lin 2014). In brief, cells were lysed with lysis buffer containing 20 mM Tris (pH 7.5), 1 mM MgCl_2_, 125 mM NaCl_2_, 1% Triton X-100, 1 mM PMSF, 10 µg/mL leupeptin, 10 µg/mL aprotinin, 25 mM β-glycerophosphate, 50 mM NaF, and 100 µM Na_3_VO_4_. Whole cell lysates (30 µg) were electrophoresed through SDS-PAGE, and the gels were transferred onto PVDF membranes. The whole membranes were blocked through incubation with 5% bovine serum albumin for 1 h. Subsequently, proteins were incubated with specific primary antibodies for 20 h at 4 °C. Then, the membranes were incubated with HRP-conjugated secondary antibody for 1 h at room temperature. Immunoreactivity was analyzed using enhanced chemiluminescence following the manufacturer’s protocol.

### Cell transfection

For transient transfection of siRNA into WI-38 cells, the transfection reagent/siRNA mixture was incubated at room temperature for 10 min and then added dropwise to MEM supplemented with 10% fetal bovine serum; it was then incubated in a humidified 37 °C incubator for 24 h.

### PEA3-luciferase activity assay

In brief, WI-38 cells were transfected with PEA3-Luc (0.8 μg) and co-transfected internal control reporter pBK-CMV-Lac Z (0.1 μg) by using transfection reagent for 24 h. Cells were stimulated with hypoxia (1% O_2_) for 18 h. Luciferase activity was measured using the luciferase assay kit (Promega, Madison, WI, USA). PEA3-luciferase luminescence was normalized to Lac Z.

### Immunohistochemistry and immunofluorescence

Lung tissues were fixed in 10% formaldehyde overnight, embedded in paraffin, and sectioned for immunohistochemistry (IHC) and immunofluorescence staining. For IHC staining, 2-μm sections were deparaffinized and then processed for antigen retrieval by using citrate buffer (pH 6.0). Endogenous peroxidase in tissue was neutralized through peroxidase blocking. The tissue was then blocked with a blocking buffer and incubated with Pref-1 and PEA3 antibodies for 40 min followed by polymer secondary antibody incubation for 30 min. Finally, the tissues were stained with hematoxylin and DAB solution. The integrated density of IHC staining was analyzed using ImageJ Fiji software. For immunofluorescence staining, 2-μm sections were deparaffinized and then processed for antigen retrieval by using EDTA buffer (pH 9.0). The tissue was blocked with a blocking buffer and incubated with Pref-1, HIF-1α PEA3, and fibronectin antibody for 24 h at 4 °C. Then, the tissue was incubated with Alexa Fluor 488-conjugated and Alexa Fluor 555-conjugated secondary antibodies for 1 h at room temperature. Images were scanned using a ScanScope CS or fluorescence microscope. The mean fluorescence intensity (MFI) of immunofluorescence staining was examined using ImageJ Fiji software.

### Immunocytochemistry

WI-38 cells were cultured on slides. After reaching confluence, cells were subjected to hypoxia (1% O_2_) for 30 min and then fixed through incubation in 4% paraformaldehyde in PBS for 10 min at room temperature. Permeabilization with PBS containing either 0.5% Triton X-100, and washed slides in PBS three times for 5 min. The coverslips were blocked with 5% bovine serum albumin in PBST for 1 h and incubated at 4 °C overnight with antibodies specific to PEA3. Cells were incubated with Alexa Fluor 488-conjugated secondary antibody for an additional 1 h. Counter staining was performed DAPI for 1 min and the coverslip was mounted with a mounting medium. They were then observed under a fluorescence microscope.

### Coimmunoprecipitation

WI-38 cells were seeded onto 10-cm dishes. After reaching confluence, cells were subjected to hypoxia (1% O_2_) for the indicated time intervals. Cells were then harvested, lysed in 100 μL of IP lysis buffer (Thermo Fisher Scientific, MA, USA), and centrifuged. The supernatant was then immunoprecipitated with a specific Ab against PEA3 (Santa Cruz, CA, USA) or c-Jun (Santa Cruz, CA, USA) in the presence of protein A/G beads at 4 °C overnight. The immunoprecipitated beads were washed three times with IP lysis buffer. The immune complex was analyzed through 8% SDS-PAGE, transferred to PVDF membranes, and then subjected to immunoblot analysis with Abs specific for serine (Abcam, Cambridge, UK), PEA3 (Santa Cruz, CA, USA), or c-Jun (Santa Cruz, CA, USA).

### Chromatin immunoprecipitation (ChIP)

WI-38 cells were subjected to hypoxia (1% O_2_) for 30 min and then fixed with 10% formaldehyde for 10 min. Cells were collected and subjected to sonication; then, anti-PEA3, or anti-c-Jun was used for immunoprecipitation, and mouse anti-IgG antibody was used as the control. The Pref-1 promoter region was amplified through the polymerase chain reaction, and the following primers were used: AP-1, 5′-ACCACGAGTCAGCTGGGTAT-3′ (sense) and 5′-TGCACACCCAAACACGCAAA-3′ (antisense) and PEA3, 5′-TTGTGTTTCAGCGCGGCTA-3′ (sense) and 5′-CAAGCGGACCTGCGGTTA-3′ (antisense). DNA was analyzed with 1% agarose gel containing ethidium bromide.

### Statistical analysis

All experimental data are presented as the mean value ± standard error of the mean for at least three independent experiments. Normality distribution was determined by Shapiro–Wilk normality test. The parametric results were conducted using one-way analysis of variance (ANOVA) followed by Dunnett’s test analysis, unpaired *t* test. nonparametric data were analyzed with Mann–Whitney *U* test. The results were considered statistically significant if *P* < 0.05.

## Results

### Pref-1 and HIF-1α expression were increased, and colocalization of PEA3 and fibronectin in lung sections from OVA-treated mice

**Fig. 1:**
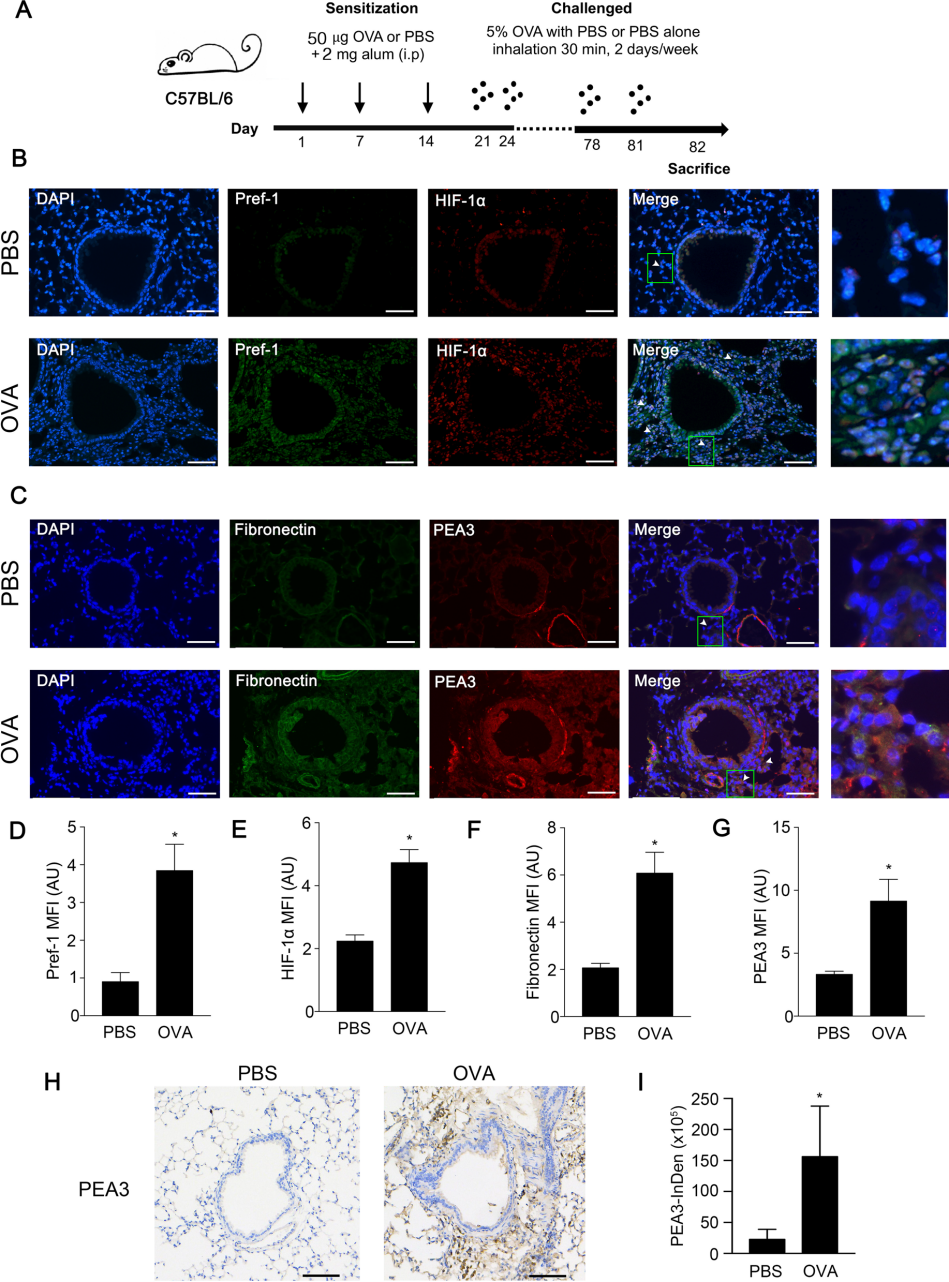
Pref-1 and PEA3 expression in lung tissue sections obtained from OVA-treated mice. **A** Mice were sensitized with OVA through three intraperitoneal injections on days 1, 7, and 14 with OVA absorbed to aluminium hydroxide. Mice were sensitized and challenged with aerosolized OVA twice weekly for up to 82 days. Control mice were treated with PBS. Lung tissue sections were stained through **B** immunofluorescence for Pref-1 (green), HIF-1α (red), **C** Fibronectin (green), and PEA3 (red) (original magnification, 20 × ; OVA n = 4, PBS n = 5). Quantification of mean fluorescence intensity (MFI) of **D** Pref-1, **E** HIF-1α, **F** fibronectin, and **G** PEA3 from **B** or **C**. Data are presented as the mean ± S.E.M., *P < 0.05, relative to the PBS group **H** immunohistochemistry for PEA3 expression (original magnification, 20 × ; OVA n = 5, PBS n = 6). **I** PEA3 Integrated density are presented as the mean ± S.E.M., *P < 0.05, compared with PBS

To evaluate Pref-1 expression in severe asthma, we used a mouse model sensitized with the allergen OVA. Mice received an intraperitoneal injection of 50 μg OVA in 2 mg aluminium hydroxide or PBS alone on days 1, 7, and 14 (Fig. 1A). These mice were challenged with aerosolized 5% OVA twice per week from day 21 to 81 and were then sacrificed for further analysis. In the OVA-sensitized mouse model, HIF-1α was increased in the nuclear, and Pref-1 expression also was detected in airway tissue by dual-label immunofluorescent staining (Fig. 1B, D, E). Moreover, we examined PEA3 expression in the lung section. Colocalization of PEA3 and fibronectin was observed through dual-label immunofluorescent staining (Fig. 1C, F, G). Increased PEA3 expression was observed in the subepithelial layer of OVA-treated mice through IHC staining (Fig. 1H, I). These results suggest that Pref-1 and PEA3 are involved in hypoxia-induced airway fibrosis in OVA-sensitized mice.

### Hypoxia-induced Pref-1 mRNA and protein upregulation in WI-38 cells

**Fig. 2:**
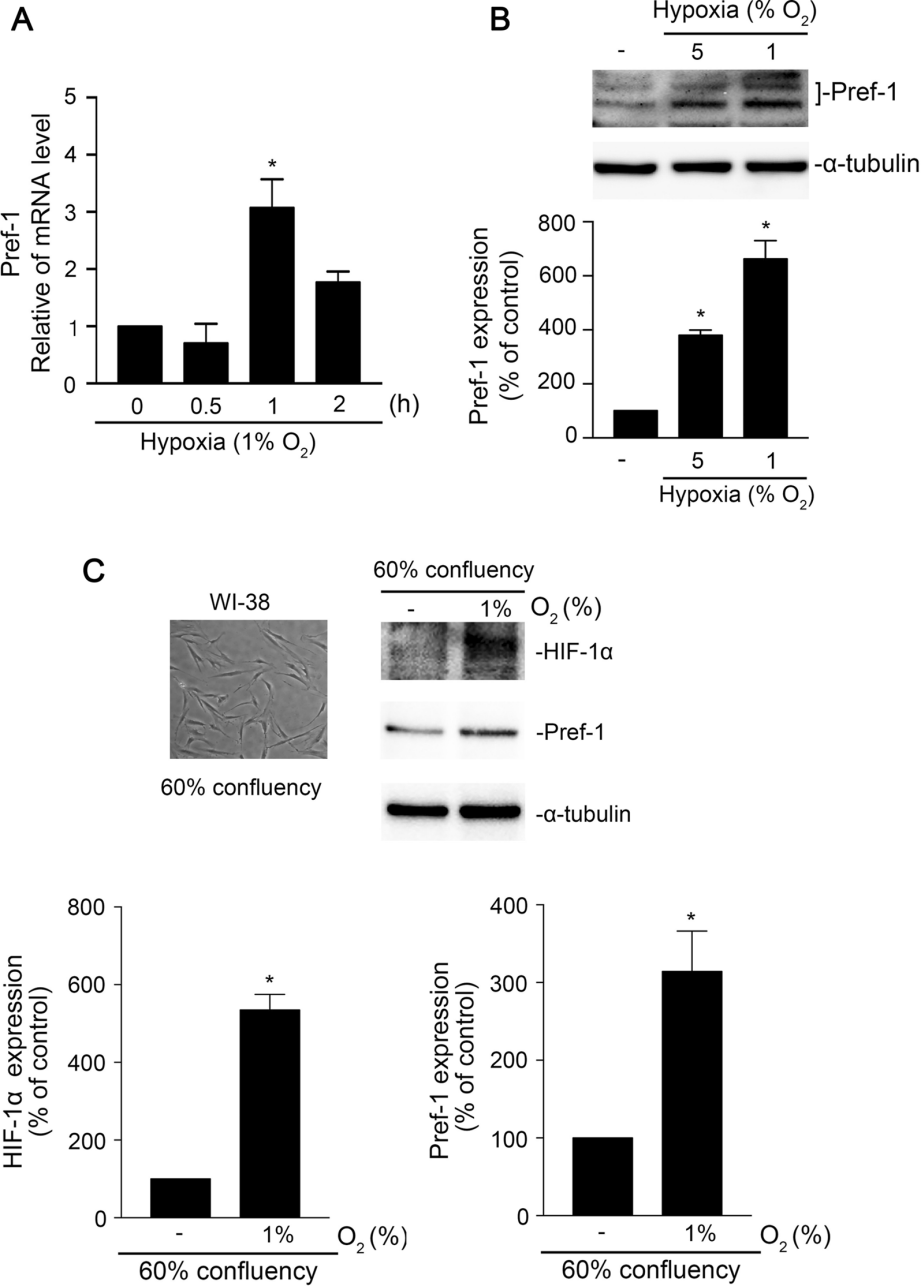
Hypoxia-induced Pref-1 expression in WI-38 cells. **A** 90% confluency WI-38 cells were incubated with 1% O_2_ for 0–2 h. The levels of Pref-1 messenger RNA were detected by using qPCR. Data are presented as the mean ± S.E.M. of three experiments. **B** 90% confluency WI-38 cells were incubated with different oxygen concentrations for 4 h, and then, Pref-1 and α-tubulin were determined through immunoblots. These are presented as the mean ± S.E.M. (n = 3). *P < 0.05, compared with control (at O_2_ 21%). **C** 60% confluency WI-38 cells were exposed with 1% O_2_ for 4 h. HIF-1α, Pref-1, and α-tubulin were detected through immunoblots. These are presented as the mean ± S.E.M. (n = 4). *P < 0.05, compared with control (at O_2_ 21%)

Hypoxia induced Pref-1 expression in preadipocytes (Moon et al. 2018). A study showed that Pref-1 is involved in human adipose tissue fibrosis (Divoux 2010). However, the mechanism by which hypoxia induces Pref-1 expression in human lung fibrosis remains unknown. We used 90% confluency of WI-38 cells before hypoxia (1% O_2_) stimulation as a cell model. Our data demonstrated that the Pref-1 mRNA level increased after hypoxic stimulation of WI-38 cells for 1 h (Fig. 2A). Furthermore, we found that Pref-1 protein expression was upregulated with a decrease in oxygen concentration (Fig. 2B). To determine effect of other cell density in hypoxia-induced HIF-1α and Pref-1 expressions. HIF-1α and Pref-1 protein expressions were increased by hypoxia (1% O_2_) in 60% confluency cells (Fig. 2C). These data indicated that hypoxia induced Pref-1 expression in 60% or 90% confluency WI-38 cells.

### PEA3 was involved in hypoxia-induced Pref-1 expression in WI-38 cells

**Fig. 3:**
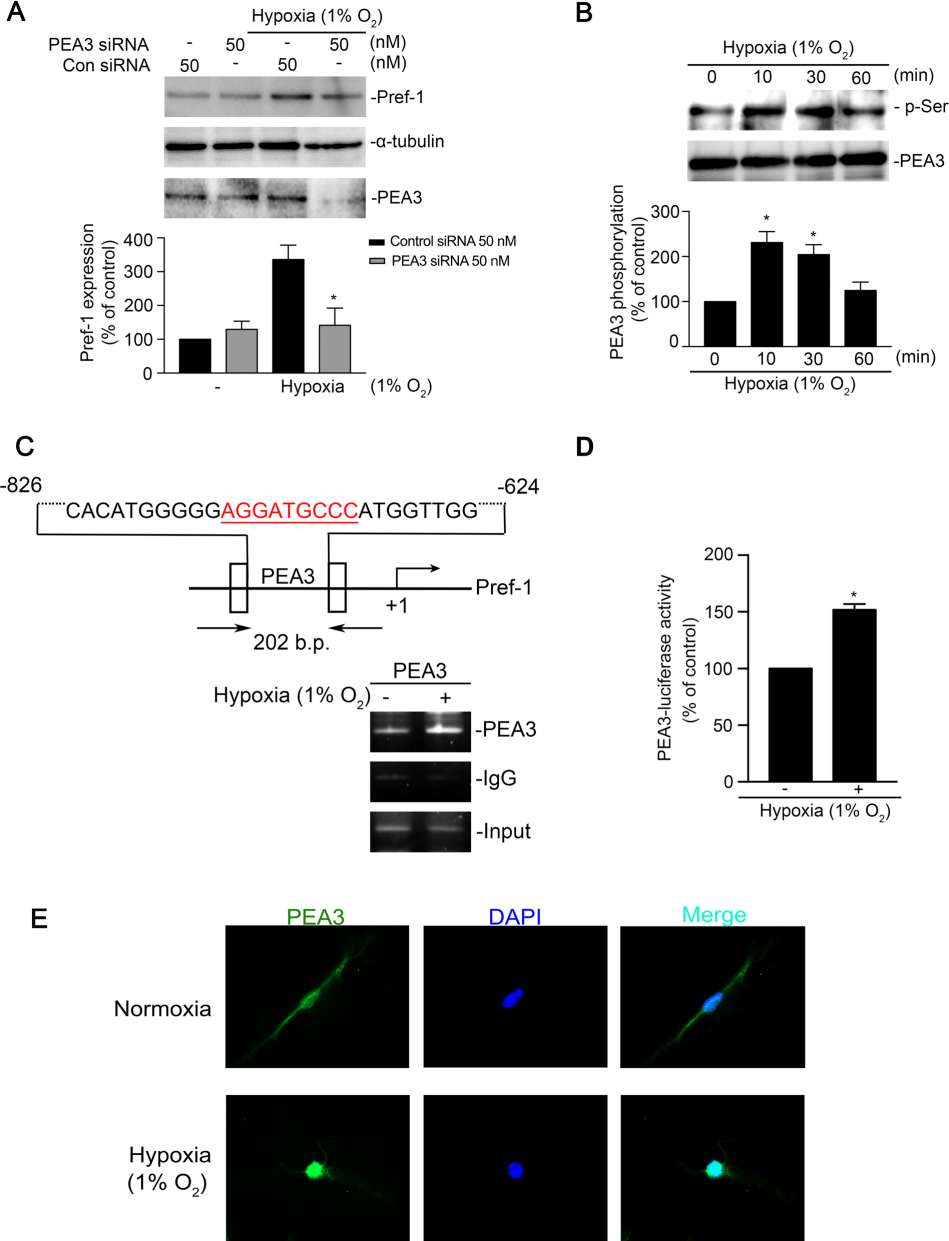
Involvement of PEA3 in hypoxia-induced Pref-1 expression in WI-38 cells. **A** WI-38 cells were transfected with control siRNA or PEA3 siRNA for 24 h and then subjected to hypoxia (1% O_2_) for another 4 h. Western blotting was performed to assess the levels of Pref-1, α-tubulin, and PEA3 in cell lysates. Data are presented as the mean ± S.E.M. of five experiments. *P < 0.05, compared with the control siRNA group. **B** WI-38 cells were subjected to hypoxia for the indicated time, and cell lysates were immunoprecipitated with an anti-PEA3 antibody; further, they were immunoblotted with antibodies for serine and PEA3. Data are presented as the mean ± S.E.M. of three experiments. *P < 0.05, compared with the control at 0 min. **C** Schematic of the PEA3 binding site located on the Pref-1 promoter. Cells were subjected to hypoxia (1% O_2_) for 30 min; the PEA3 binding site of the Pref-1 promoter region was detected through the ChIP assay. Input for for use as a positive control. Mouse polyclonal IgG for use as a negative control. Traces indicate that the three experiments produced similar results. **D** Cells were transfected with 0.8 μg of PEA3-Luc and 0.1 μg of pBK-CMV-Lac Z for 24 h and then subjected to hypoxia (1% O_2_) for another 16 h. Cells were harvested for a luciferase activity assay. Data are shown as the mean ± S.E.M., n = 3. * *P* < 0.05, relative to nonstimulated cells. **E** WI-38 cells were subjected to hypoxia (1% O_2_) for 30 min. In confocal microscopy, the cells were incubated with antibodies specific for PEA3, and immunoreactivity was performed through the incubation of the cells with an FITC-conjugated secondary antibody. All slides were counterstained with DAPI (blue) to distinguish the nucleus, which were visualized under an immunofluorescence microscopy (original magnification, 20 × ; n = 3)

To determine the role of PEA3 in hypoxia-induced Pref-1 expression in human lung fibroblasts, WI-38 cells were treated with PEA3 siRNA (50 nM) overnight and then subjected to hypoxia (1% O_2_). We found that hypoxia-induced Pref-1 expression significantly decreased after PEA3 siRNA transfection (Fig. 3A). Moreover, we exposed WI-38 cells to hypoxia for various time intervals. PEA3 serine phosphorylation was observed at 10 min, which declined after 60 min of exposure (Fig. 3B). We used the NCBI database to predict the transcription factor binding site in the promoter region of Pref-1; we found that PEA3 and AP-1 are the binding sequences in the promoter region of Pref-1. Furthermore, the ChIP assay showed PEA3 binding to the promoter region of Pref-1 in WI-38 cells during hypoxia (Fig. 3C). Hypoxia-stimulated cells showed a increase in PEA3-luciferase activity at 24 h (Fig. 3D). PEA3 was translocated to the nucleus from the cytoplasm after hypoxia in WI-38 cells, as revealed by immunocytochemistry (Fig. 3E). Taken together, these data indicated that PEA3 activation was involved in hypoxia-induced upregulation of Pref-1 expression.

### Involvement of ERK phosphorylation in PEA3 and AP-1 activation for hypoxia-induced Pref-1 expression in WI-38 cells

**Fig. 4:**
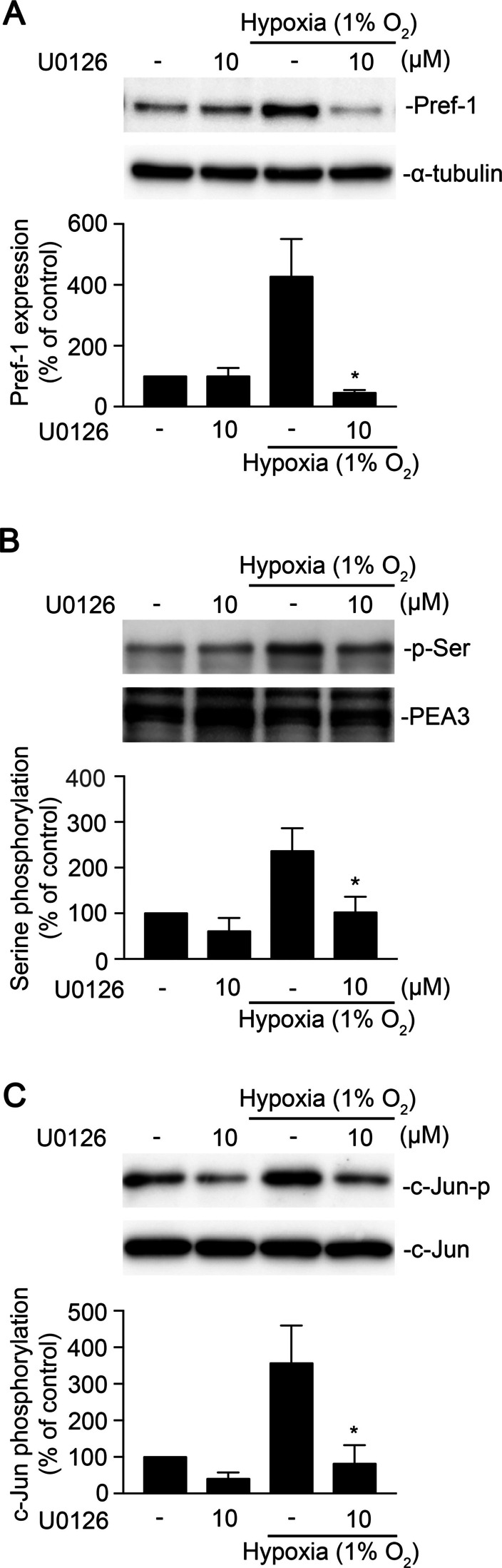
Involvement of ERK in hypoxia induces a Pref-1 expression in WI-38 cells. **A** WI-38 cells were pretreated with U0126 for 30 min and then incubated with the hypoxia (1% O_2_) for 4 h. Levels of Pref-1 and α-tubulin in cell lysates were determined. Data are presented as the mean ± S.E.M. of three experiments. * *P* < 0.05, compared with the hypoxia-exposed group. Cells were pretreated with U0126 for 30 min and then subjected to hypoxia (1% O_2_) for 30 min. **B** PEA3 was immunoprecipitated using anti-PEA3 antibody, and PEA3-p-serine was detected using anti-p-serine antibody. The quantified results were adjusted with PEA3 and expressed as a percentage of control. **C** Levels of phospho-c-Jun Ser63 and c-Jun were detected using Western blotting in cell lysates. Values represent the means ± S.E.M. of three experiments. * *P* < 0.05 compared with the hypoxia-exposed group

PEA3 plays a role in hypoxia-induced Pref-1 expression. A study indicated that ERK plays a crucial role in PEA3 activation in gastric adenocarcinoma (Keld 2011; Guo and Sharrocks 2009). We aimed to elucidate whether hypoxia induced Pref-1 expression through ERK/PEA3/AP-1 signaling in WI-38 cells. Treatment of cells with U0126 (10 μM), an ERK inhibitor, downregulated hypoxia-induced Pref-1 expression (Fig. 4A). Moreover, U0126 downregulated hypoxia-induced PEA3 and c-Jun phosphorylation in WI-38 cells (Fig. 4B, C). These results demonstrated that ERK mediated hypoxia-induced Pref-1 expression, PEA3 and c-Jun phosphorylation in WI-38 cells.

### Formation of the AP-1/PEA3 complex mediated Pref-1 gene expression in WI-38 cells

**Fig. 5:**
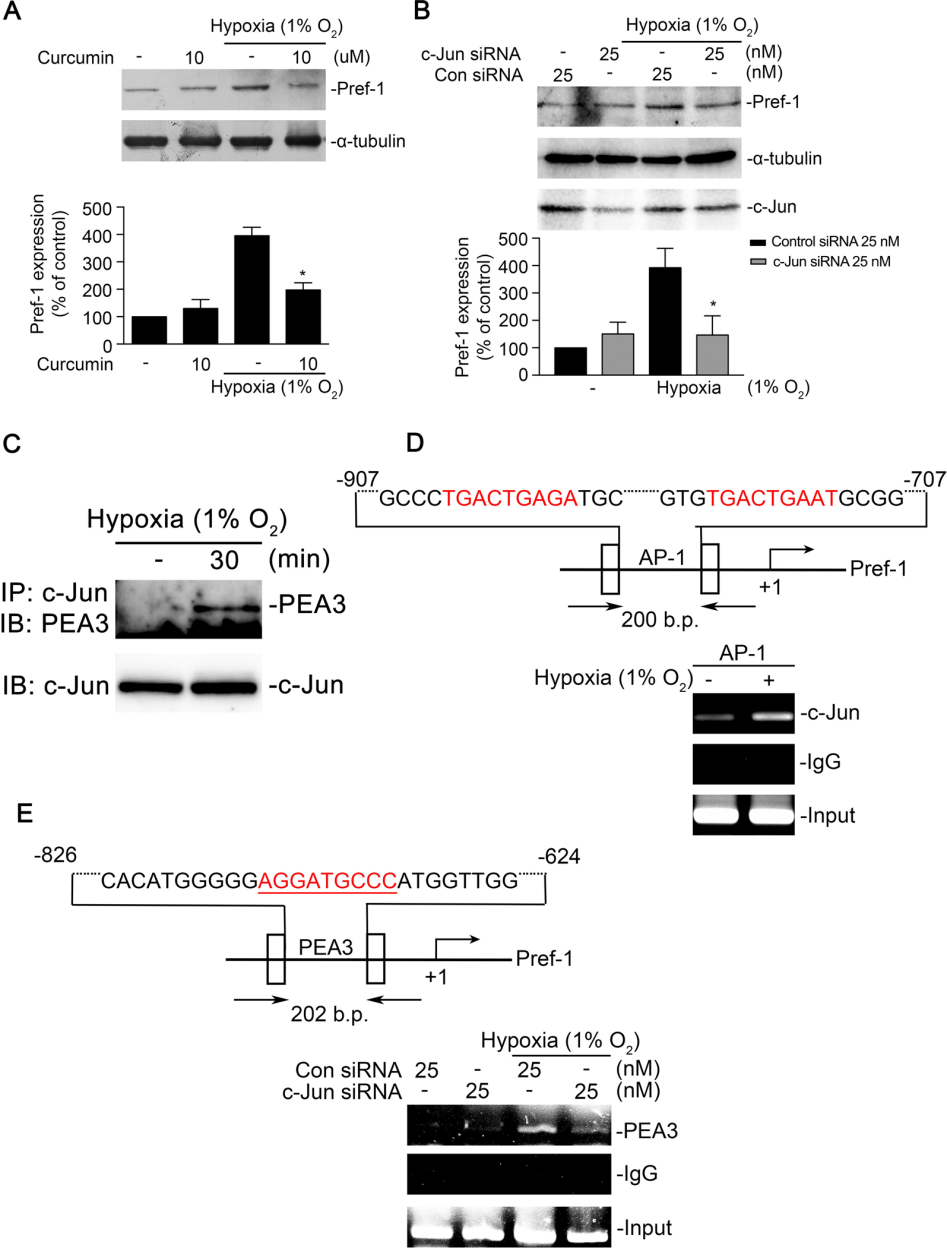
Involvement of AP-1 in hypoxia-induced Pref-1 expression in WI-38 cells. **A** Cells were pretreated with curcumin (10 μM) for 30 min and then stimulated with hypoxia (1% O_2_) for another 4 h. Pref-1 and α-tubulin were detected in cell lysates. Data are presented as the mean ± S.E.M. for three independent experiments. *P < 0.05, compared with the hypoxia group. **B** WI-38 cells were transfected with control siRNA or c-Jun siRNA for 24 h and then subjected to hypoxia (1% O_2_) for another 4 h. Western blotting was performed to assess the levels of Pref-1, α-tubulin, and c-Jun in cell lysates. Data are presented as the mean ± S.E.M. of five experiments. *P < 0.05, compared with the control siRNA group. **C** WI-38 cells were exposed to hypoxia (1% O_2_) for 30 min. Cells were lysed with IP lysis buffer and then immunoprecipitated with the anti-c-Jun antibody. The immunoprecipitated complex was detected through immunoblotting with an anti-PEA3 antibody. Typical traces were demonstrative of three experiments. **D** Schematic of the AP-1 binding site located on the Pref-1 promoter. WI-38 cells were exposed to hypoxia (1% O_2_) for 30 min. The AP-1 binding site of the Pref-1 promoter region was detected through ChIP assay. Typical traces were presented in all three experiments. **E** Schematic of the PEA3 binding site located on the Pref-1 promoter. After transfection with c-Jun siRNA for 24 h, cells were subjected by hypoxia for 30 min, The PEA3 binding site of the Pref-1 promoter region was detected through ChIP assay. Typical traces were presented in all three experiments

A study showed that the AP-1/PEA3 complex binds to *IL-8/CXCL8* promoter in human hepatocellular carcinoma (Iguchi 2000). However, the AP-1/PEA3 complex is involved in hypoxia-induced Pref-1 expression remains unknown. In this study, curcumin (10 μM), an AP-1 inhibitor, and c-Jun siRNA (25 nM) attenuated hypoxia-induced Pref-1 expression in WI-38 cells (Fig. 5A, B). Moreover, PEA3 antibodies coprecipitated PEA3 and c-Jun after exposure to hypoxia in WI-38 cells (Fig. 5C). We observed that AP-1 bound to the promoter of Pref-1 after hypoxia exposure in the ChIP assay (Fig. 5D). Also, c-Jun siRNA (25 nM) down-regulated the binding of PEA3 to the Pref-1 promoter (Fig. 5E). Collectively, the results showed that the AP-1/PEA3 complex was involved in Pref-1 expression in WI-38 cells.

## Discussion

The present study demonstrated that hypoxia induces ERK phosphorylation, which in turn activates PEA3 and AP-1 that binds to the promoter region of Pref-1; this upregulates Pref-1 expression in human lung fibroblast cells. These results indicate that hypoxia-induced Pref-1 may play a crucial role in airway fibrosis.

In fibroblastic lung diseases, fibroblast proliferation, and differentiation can rapidly decline lung function (King et al. 2011; Mizuno 2009). Studies have demonstrated that moderate to severe hypoxia (0.1–5%) can promote the proliferation of airway smooth muscle cells and lung fibroblasts (Ahmad 2012; Mizuno 2009; Cogo 2003). However, the correlation of hypoxia and Pref-1 with airway fibrosis remains unknown. Previous studies showed that hypoxia-induced gene expression by the up-regulation of HIFs (Bahrami et al. 2018). HIFs is a heterodimer protein consisted of one of α-subunits (HIF-1α, -2α, or -3α) and two constitutive β-subunit (HIF-1β or ARNT2) (Strofer et al. 2011). Under the normoxia environment, HIF-1α would be hydroxylated and degraded. However, hypoxia can prevent HIF-1α from degradation and then form a stable complex with HIF-1β to activate downstream gene expression (Bahrami et al. 2018). The structure of HIF-2α is similar to HIF-1α and also forms a stable complex with HIF-1β under hypoxic state (Bahrami et al. 2018). Moreover, HIF-1α and HIF-2α could bind to hypoxia-responsive elements (HREs] in the promoter of Pref-1 and increased Pref-1 transcription in cancer cells (Kim et al. 2009b). In contrast, the function of HIF-1α and HIF-2α is opposite in some physiological processes (i.e., angiogenesis and erythropoietin production) (Loboda et al. 2012; Bjørås 2019). In the present study, nuclear HIF-1α expression and Pref-1 expression were increased in the OVA-induced allergic airway. Previous studies showed that HIF-1α was increased in confluence-dependent manner in many cells (Fang et al. 2007; Sheta et al. 2001), there were evidences that confluent cells could cause reduction of pO_2_ and pericellular hypoxia. In our study, hypoxia also induced HIF-1α and Pref-1 expression in 60% confluency WI-38 cells. It can be implied that Pref-1 is associated with the hypoxia environment in airway fibrosis. However, the mechanism of Pref-1-induced lung fibroblast differentiation requires further investigation.

Pref-1 suppressed adipogenesis by binding to the integrin receptor and then inhibited C/EBPβ and C/EBPδ genes in preadipocytes (Wang and Sul 2009). Pref-1 showed strong correlations with various metabolic conditions, including hepatic steatosis, blood pressure, and insulin sensitivity (O'Connell et al. 2011). Moreover, Pref-1 impaired proinflammatory cytokine expression in human bone marrow mesenchymal stem cells (Abdallah 2007). These findings suggest that Pref-1 is involved in chronic inflammation and metabolic diseases. A study reported that hypoxia induces Pref-1 expression in preadipocytes during adipogenesis but does not increase Pref-1 levels in adipocytes. It may hypoxia alters histone modification in preadipocytes (Moon et al. 2018). In this study, hypoxia upregulated Pref-1 mRNA and protein expression through the ERK/PEA3/AP-1 cascade in human lung fibroblasts.

A study showed that PEA3 is involved in many processes, including epithelial mesenchymal transition, apoptosis, cell invasion, and chemotherapy resistance (Qi 2020). A study showed that mitogen-activated protein kinase (MAPK) signaling upregulated PEA3 expression (Keld 2011). Moreover, PI3K/Akt signaling activated PEA3 expression in renal cell carcinoma (Xu 2020). In this study, PEA3 was activated with ERK, and then, it bound to the Pref-1 promoter region after hypoxia exposure in WI-38 cells. We found that hypoxia induced PEA3 phosphorylation and translocation to the nucleus, which in turn induced Pref-1 expression in WI-38 cells. Furthermore, PEA3 siRNA downregulated hypoxia-induced Pref-1 expression. These results demonstrated that PEA3 contributed to hypoxia-induced Pref-1 expression.

MAPK/ERK regulates lung fibrogenesis and cell growth and proliferation (Madala 2012). Several studies have reported that ERK is essential for mediating profibrotic gene expression through the activation of transcriptional factors, including AP-1 (Lin 2014; Engers 2006). MAPK/ERK regulates transforming growth factor β1-induced human alveolar type II cell senescence and epithelial mesenchymal transition (Chen 2020). Moreover, hypoxia induces ERK Tyr204 phosphorylation, which in turn contributes to AP-1 activation and connective tissue growth factor expression in WI-38 cells (Cheng 2017). In the present study, ERK was involved in hypoxia-induced PEA3 phosphorylation, c-Jun phosphorylation and, Pref-1 expression in human lung fibroblasts. Thus, hypoxia induced Pref-1 expression through the ERK/PEA3/AP-1 pathway.

A study demonstrated that hypoxia induced AP-1 expression and promoted downstream gene expression (You et al. 2010). A study showed that hypoxia induced AP-1 phosphorylation and contributed to fibrogenic protein expression in human lung fibroblasts (Cheng 2017). In retinal vascular endothelial cells, AP-1 activated JNK under hypoxia and then induced Cyr61 protein expression (You et al. 2010). Previous studies demonstrated that curcumin affected HIFs in tumor cell proliferation and metabolic diseases, e.g., curcumin decreased growth and survival of hepatoma, and breast carcinoma cells through degradation of HIF-1α and HIF-2α (Bahrami et al. 2018; Strofer et al. 2011). Zhao et al. reported that curcumin suppressed collagen and α-SMA by down-regulation of ERK/HF-1α expression in hematopoietic stem cells of rat (Zhao 2014). Moreover, curcumin attenuated adipose fibrosis through inhibition of AMPK/mTOR/HIF-1α signaling (Qiu 2017). Take together, curcumin prevented hypoxia-induced HIFs activation in fibrosis, cancers, and metabolic diseases. Here, we demonstrated that curcumin inhibited hypoxia-induced Pref-1 expression in human lung fibroblasts. Moreover, c-Jun siRNA down-regulated Pref-1 expression, and the AP-1/PEA3 complex was recruited to the Pref-1 promoter after hypoxia in human lung fibroblasts.

## Conclusion

**Fig. 6:**
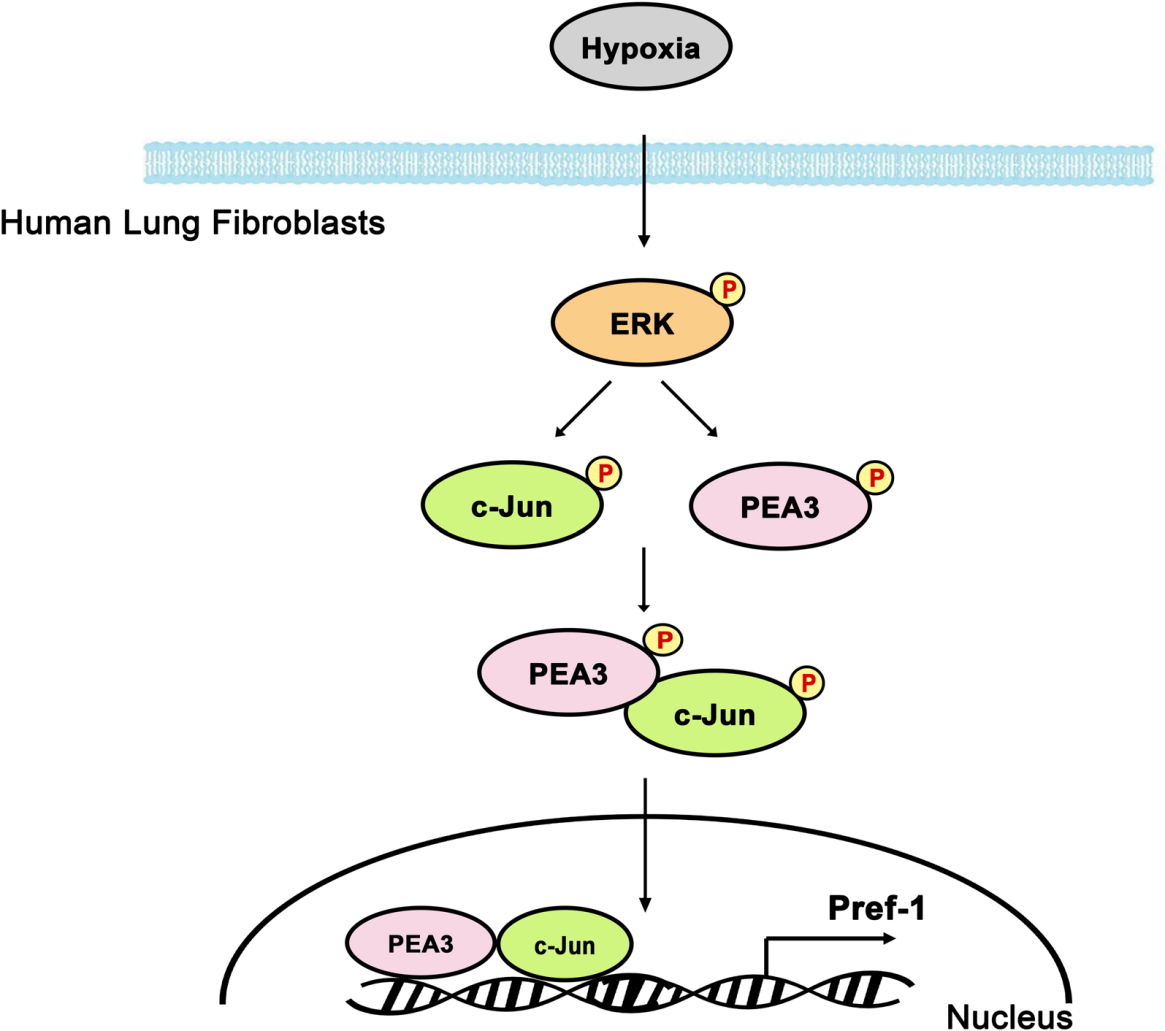
Simplified image displaying the results of the expression of hypoxia-induced Pref-1 through the ERK/PEA3/AP-1 pathway in human lung fibroblasts. Hypoxia induced the activation of ERK, which in turn caused PEA3 and AP-1 phosphorylation and complex formation. Moreover, PEA3 and AP-1 complex mediates Pref-1 expression through hypoxia stimulation in human lung fibroblasts

In conclusion, these findings showed that hypoxia activates Pref-1 expression through the ERK/PEA3/AP-1 signaling pathway. Figure 6 shows a simplified diagram of the signaling pathway that demonstrates hypoxia-induced Pref-1 upregulation in human lung fibroblasts. Our findings demonstrate how hypoxia induces Pref-1 expression in human lung fibroblasts.

## Data Availability

Not applicable.

## Abbreviations

ADAM17: A disintegrin and metalloproteinase 17
AP-1: Activator protein 1
ChIP: Chromatin immunoprecipitation
ERK: Extracellular signal-regulated kinase
ETs: Erythroblast transformation specific
FITC: Fluorescein isothiocyanate
HIF: Hypoxia-inducible factor
HREs: Hypoxia-responsive elements
HRP: Horseradish peroxidase
IgG: Immunoglobulin G
IHC: Immunohistochemistry
MAPK: Mitogen-activated protein kinase
MEM: Minimum essential medium
MFI: Mean fluorescence intensity
NCBI: National Center for Biotechnology Information
OVA: Ovalbumin
PBS: Phosphate-buffered saline
PEA3: Polyoma enhancer activator 3
Pref-1: Preadipocyte factor 1
siRNA: Small interfering RNA
